# How Accurate Can 2D LiDAR Be? A Comparison of the Characteristics of Calibrated 2D LiDAR Systems

**DOI:** 10.3390/s25041211

**Published:** 2025-02-17

**Authors:** Adam Ziębiński, Piotr Biernacki

**Affiliations:** 1 Department of Distributed Systems and Informatic Devices, Silesian University of Technology, Akademicka 16, 44-100 Gliwice, Poland; 2 Institute of Theoretical and Applied Informatics, Polish Academy of Sciences, Bałtycka 5, 44-100 Gliwice, Poland; pbiernacki@iitis.pl

**Keywords:** AGV, 2D LiDAR, curve fitting, filtration, sensor calibration, single-beam distance sensors

## Abstract

The utilization of 2D Light Detection and Ranging (LiDAR) measurements does not always provide the precision needed to accurately determine the motion range or recalibrate the position of Autonomous Guided Vehicles (AGVs). Consequently, it is essential to employ filtering and calibration methods to enhance the precision and accuracy of measurements derived from 2D LiDAR. The article proposes a multi-sectional calibration (MSC) method incorporating a median filtration (MF) phase to enhance the measurement accuracy of 2D LiDAR. The investigation focused on identifying the optimal window width for the MF module among a selection of 2D LiDAR systems. The division of the complete measurement range into sections resulted in a significant enhancement in sensitivity to deviations in measurements. The efficacy of the proposed method is evidenced by its ability to enhance accuracy in distance measurements by up to 89% for the optimal window width. The experiments indicated that the proposed method has a significant impact on the precision and accuracy of distance measurements for 2D LiDAR systems.

## 1. Introduction

Additional solutions are now being developed for modern production systems [1], especially to increase the productivity of robotic assembly lines [2], which increasingly use collaborative robots (CRs) [3] and internal transport systems (ITSs) [1,4].

AGVs, which are often used as ITSs, enable cooperation with production personnel and production infrastructure, including automated production stations, automated warehouses, assembly stations (ASs), robots [5], or CRs [6]. AGV logistics tasks support the production process [7] and should be carried out in a dynamic, autonomous manner that allows for accurate arrival and docking at destination points. Precise position determination, especially for moving staff, CR, and AGVs, is a crucial factor for safety assurance in the production plant [8]. Determining the exact location [9] of detected objects is a prerequisite for ensuring the accurate operation of the navigation system for an AGV [10] and calculating the repositioning of a robotic arm. To make this possible, precise monitoring [11] of AGVs and CRs in an industrial environment [12] is necessary. To ensure control of the movements [13] of such systems, they are equipped with an increasing number and variety of sensors [14].

Among the many factors analyzed by AGV systems, distance measurements [15] are important to ensure the correctness [16] and accuracy of the tasks performed in an industrial environment. Ultrasonic sensors [17], LiDAR from 1D to 3D [18], radars, UWBs [10], and mono and stereo cameras [19] can detect and determine the distance to an obstacle [20], thereby localizing and avoiding it [21]. The laser sensors use common distance measurement methods, triangulation [22], and time-of-flight (ToF) [23]. Two-dimensional LiDAR [24] has been proven to facilitate the sensing of the surrounding environment and to provide high-frequency measurements of distance, velocity, and reflectivity.

The utilization of AGV systems in the vicinity of production lines operated by the CR systems and staff requires ensuring safe and accurate driving [25]. Consequently, 2D LiDAR is most commonly employed for distance measurements, which ensures the safety and proper implementation of autonomous functionality such as moving between indicated points, platooning, docking [26], loading [27], charging, and cooperating with production staff [28], the CR, and the assembly station (AS).

**Figure 1:**
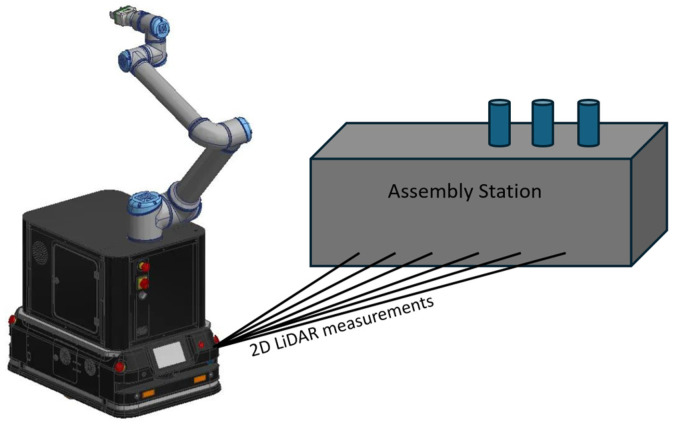
An example of the measurement of distance to the AS using a 2D LiDAR sensor mounted on the CoBotAGV [30].

High-precision distance measurements [29] are particularly relevant in the context of ensuring accurate docking of the AGV to the AS, as well as at intermediate points where the AGV must halt to recalibrate its position. For instance, following the docking of the AGV to the AS, the CR mounted on the AGV (CoBotAGV) [30] will acquire information regarding the precise and accurate position of the AGV relative to the AS (Figure 1). This facilitates accuracy enhancement during the recalibration of the CR arm to its new mounting position on the AS. Furthermore, the CR can assess whether the docking location allows for the proper and safe performance of assembly tasks on the AS.

For this reason, it is important to acquire accurate measurements from 2D LiDAR sensors [31] to determine the range of motion and provide more accurate recalibration of the position of the AGV [32] and other subsystems, such as the CR arm.

In effect, various calibration methods [33], including curve fitting [34], filtering [35], and data fusion [36], are required.

The purpose of this study was to improve the overall measurement accuracy of 2D LiDAR distance sensors. Our aim was to develop a method for calibrating measurements based on multi-section curve fitting with a filter phase for single-beam distance sensors used in 2D LiDAR. The novelty of the proposed solution is the use of a filter phase with a specified window width for the multi-section curve fitting method. The presented method enables the selection of the required accuracy of the distance measurement for a given 2D LiDAR sensor based on the choice of the filter window width. The proposed method will enable more accurate verification of the AGV’s position relative to the assembly station, thereby ensuring the correct implementation of logistic tasks carried out by robotic systems.

The paper is organized as follows: in the second Section, the results of the investigation of the distance characteristics of selected 2D LiDAR sensors are presented; the third Section describes the proposed multi-section calibration methods with the filtration phase; the fourth Section presents the research results of the distance calibration for the selected 2D LiDAR sensors; and conclusions are presented in the fifth Section.

## 2. The Accuracy Characteristics of Selected 2D LiDAR Distance Measurements

To determine whether the proposed method is effective for different 2D LiDAR classes, the Leuze RSL425 XL [37] and SICK microScan3 [38] safety laser scanners and RPLiDAR A2 [39] and RPLiDAR A1 [40] laser range scanners were selected for experimental verification of distance measurement accuracy.

Each sensor has different features (Table 1): reflectivity sensitivity, operating range, resolution, accuracy, angular resolution, and field of view (FOV). Typically, 2D LiDAR uses the rotational motion of a laser sensor to scan a given FOV and provide measurement information in the form of the distance for a given 2D polar angle. The tested measurement range was limited to 2.25 m owing to the docking procedure requirements of the CobotAGV project.

The RSL425 XL and microScan3 safety 2D LiDAR scanners share similar characteristics. They both possess angular resolutions of 0.1 degrees and distance measurement resolutions of ±0.001 m. The RSL425 XL covers a field of view of 270 degrees. Its measurement range extends up to 50 m, with a minimum measurable distance of 0.05 m and measurement error up to 0.02 m. In comparison, the microScan3 offers a slightly wider field of view (275 degrees). Its measurement range also extends up to 64 m, with a minimum measurable distance of 0.1 m and a measurement error of up to 0.025 m. Both the RPLiDAR A1 and A2 range 2D LiDAR scanners cover a 360-degree field of view. They have a resolution below 0.5 mm for distances under 1.5 m and 1% above 1.5 m, with a measurement error below 1%. The RPLiDAR A1 offers 1 degree of angular resolution and distance measurements from 0.15 to 12 m. On the other hand, the RPLiDAR A2 offers 0.9 degrees of angular resolution and distance measurements from 0.15 to 16 m. The Leica DISTO X4 [41], which is certified (ISO 16331-1) [42] for measurement accuracy of 1 mm, was selected as the reference sensor for distance measurements. It can measure distances using a laser beam with a field of view of 0.0344 degrees, from 0.05 to 150 m with a resolution of 0.1 mm and error measurement of 1 mm at 0.05 to 10 m.

**Figure 2:**
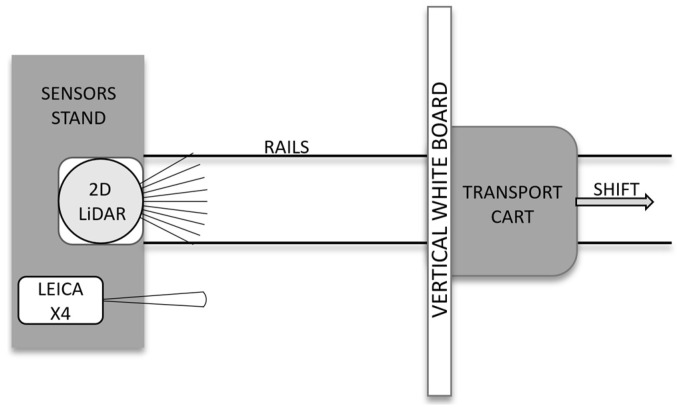
The test stand scheme is used to study the distance measurement characteristics of the 2D LiDAR sensors.

In the initial phase of the study, the distance measurement characteristics of selected 2D LiDAR sensors were investigated. A test stand was constructed to conduct precise distance measurements using the LiDAR sensors (Figure 2). The test stand consisted of the following components: a sensor stand, a transport cart, rails on which the cart was moved, and a white surface board on the cart. All experiments were done by mounting the selected 2D LiDAR and Leica sensors on the sensor stand and then measuring the distance to the white surface board, which was manually moved on a transport cart.

The cart with the obstacle was placed at a starting position from the sensor stand, which was 0.075 m for the RSL425 XL, 0.1 m for the microScan3, and 0.15 m for the RPLiDAR sensors. The prepared application then began recording 40 measurements, with each measurement recorded at equal time intervals (300 ms). When all the measurements from a single series were recorded, the cart with the board was moved on the rails by 0.05 m to a distance of 2.25 m.

**Figure 3:**
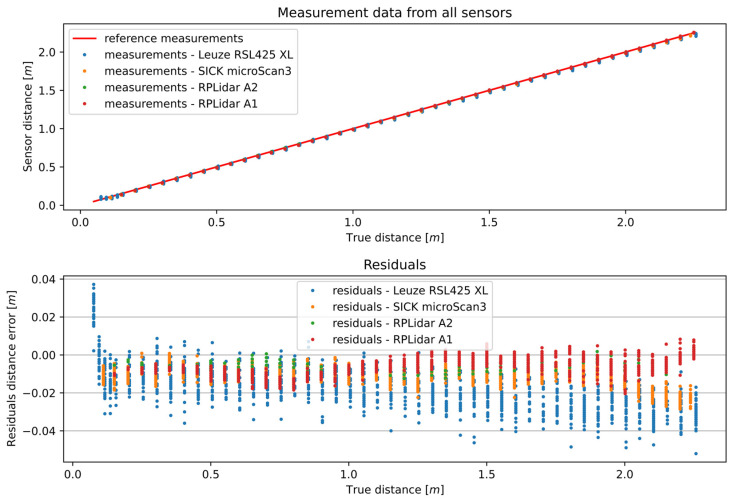
Results of the distance measurements of all the 2D sensors and their residuals.

The tested 2D LiDAR sensors were configured using default settings and were not programmatically corrected in any manner to allow for a comparison of their characteristics. The results of the distance measurements of all the 2D LiDAR sensors are presented in Figure 3. In the upper part of the figure, the distance measurements for the uncalibrated sensors and the reference sensor are presented. In the lower part, the differences between the distance measurements of the uncalibrated sensors and the reference sensor are presented. As can be observed, the measurement error for each sensor is different for each measured distance. This affects the accuracy of the distance measurement at any distance from the target. The sensors must, therefore, be calibrated to achieve greater accuracy.

**Figure 4:**
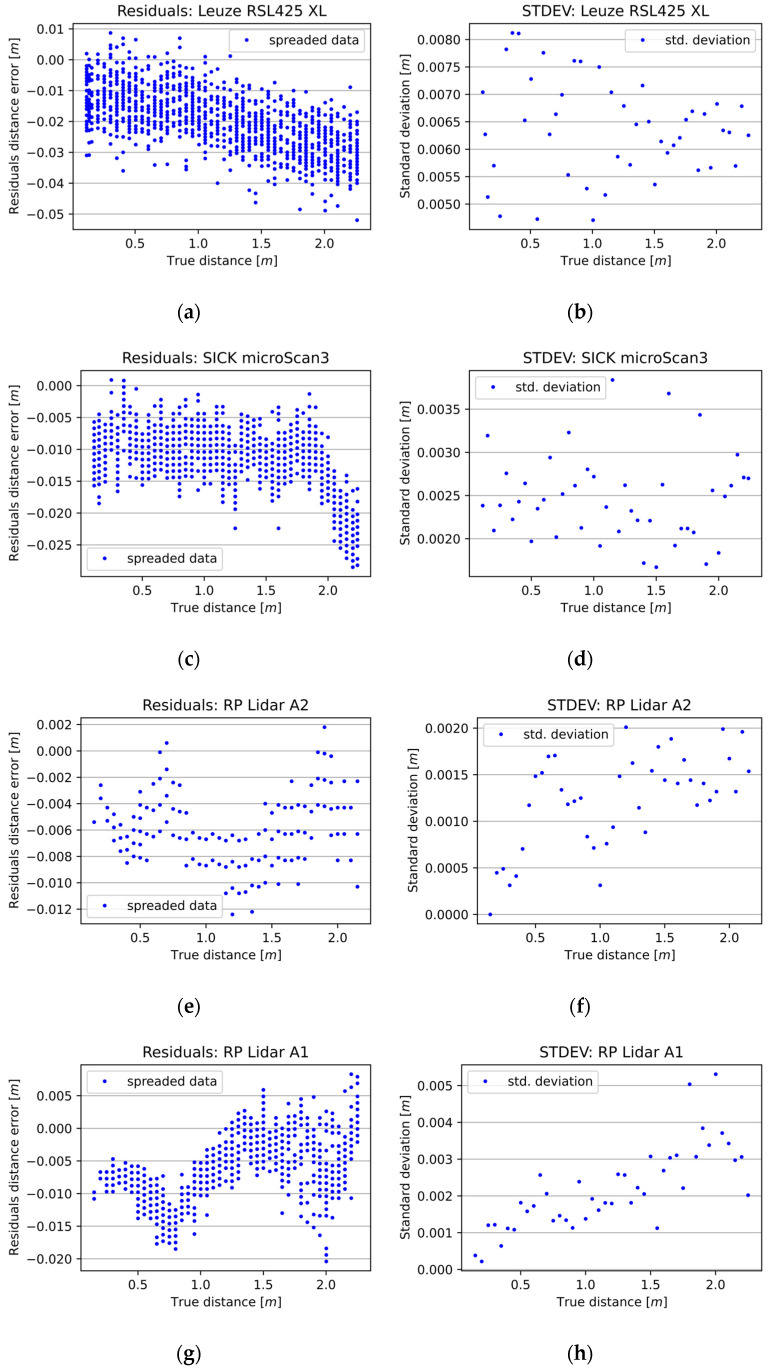
The measurement characteristics of selected 2D LiDAR sensors. The left column (**a**,**c**,**e**,**g**) presents the differences between the 2D LiDAR sensors’ measurements and the reference distance measurements. The right column (**b**,**d**,**f**,**h**) presents the standard deviation of a given sensor at a given distance.

Owing to the high measurement errors obtained at the beginning of the measurement range of the RSL425 XL, the measurement series of the RSL425XL selected for analysis starts from a distance of 0.115 m. This adjustment was necessary because, within the initial range, the measurement errors exceeded the distance by which the transport cart with the whiteboard was moved. An overview of the measurement characteristics of the tested 2D LiDAR sensors is shown in Figure 4. As can be seen, the accuracy decreased irregularly with the distance between the 2D LiDAR device and the measured object.

The distance measurement quality values obtained from the individual sensors are presented in Table 2. The standard deviation (STDEV) describes the sensor’s precision, and the residual values represent the overall sensor accuracy. STDEV values were first calculated across the given measurement series, and then the minimum, maximum, and average values of STDEV were chosen from the entire measurement range of a given sensor. The difference between the maximum and minimum of the STDEV indicates the spread across the entire measurement range. Minimum, maximum, and average values of residuals were also calculated and chosen from the entire measurement range of the given sensor.

Based on the 2D LiDAR measurement results obtained, the microScan3 proved to be the best safety scanner, with an accuracy of 0.0124 m and a precision of 0.0025 m. In contrast, the accuracy of the RSL425 XL was 0.022 m, and the precision was 0.0064 m. The most precise and accurate sensor of the 2D distance sensors was the RPLidar A2 with an RMSE equal to 0.0064 m and an average STDEV of 0.0012 m. The RPLidar A1 had slightly worse precision (0.0022 m) and accuracy (0.0079 m).

In essence, the STDEV value was predominantly above 0.002 m, with one instance exceeding 0.001 m. In the case of safety solutions, the sensors had much better angular resolution and scanning frequency properties, allowing a much more detailed representation of the scanned scene or object. However, these results may be insufficient for applications requiring greater precision and accuracy. So, there remains scope for improvement in the quality of the measurements obtained by the 2D LiDAR sensors currently available.

## 3. The Calibration Method

Calibration is used to increase the measurement accuracy of the distance sensor [33], which is the process of comparing measurements and matching them to actual values. One of the basic methods is nonlinear regression based on measurements from reference and tested sensors [34].

Another method is curve fitting [43], which is an iterative process of linear and nonlinear approximation. For curve fitting, the model that best fits the data is selected, e.g., the polynomial, exponential, power series, least square [44], or robust regression [45] method. The calibration system for ToF sensors that analyses the intrinsic properties determines correlated data and considers the reflectance of the object is presented in the paper by Lindner et al. [33].

An additional distance sensor to calibrate the mechanical displacement of the equipment was used in the work of Jin et al. [46]. Several solutions are currently used for a multi-sensor system with LiDAR and a camera for survey analysis and calibration [47]. Nevertheless, the precise and accurate measurements generated by the LiDAR remain crucial, as they have a great impact on obtaining accurate measurements by the whole system.

In this paper, a multi-sectional calibration method incorporating a median filtration phase (MSCwMF) for 2D LiDAR sensors is proposed. The multi-section curve fitting is preceded by a median filtering phase for RAW measurements. The MF can be realized with different window widths.

The result is correction data for a given 2D LiDAR sensor and for a given width of the filter window. The specific correction data set may be employed, contingent on the selected speed of the AGV and the capacity to select the angular velocity of the particular LiDAR.

### 3.1. Problem Definition

As Figure 4 shows, every 2D LiDAR sensor has its own measurement characteristics. Their common property is that they lack accuracy and precision. It is possible to calibrate them using a number of calibration methods, but this will only improve the accuracy of the given sensor. The precision of the measurements also influences the final accuracy of the given measurement system, and the filtration phase helps the curve fitting process, narrowing the spread of the measurements in the series. This is the reason for using the filtration phase prior to the calibration method.

### 3.2. Multi-Section Calibration Method with Median Filtration

The MSCwMF narrows the spread of the sensor measurements and curve fits a set of regression functions into the sensor measurements to mimic the characteristics of the sensor measurements and precisely correlate the sensor’s measurement error with the measurement it perceives.

The method consists of the following steps:

1. Raw measurement filtration using MF with a given window width;
2. Calculation of averages from each series of measurements of the reference sensor and the one under calibration;
3. Residuals calculation—differences between the reference sensor and the one under calibration;
4. Search for local minima and maxima points of residuals to determine the descending and ascending sections;
5. For each determined section,
  a. Curve fitting to reference sensor measurements;
  b. Evaluation of curve fitting efficacy to the reference sensor measurements;
  c. Curve fitting to measurements of the sensor under calibration;
  d. Evaluation of curve fitting efficacy to the sensor under calibration;
  e. Residuals calculation—differences between the reference regression line and the curve fitted to the sensor under calibration;
  f. Curve fitting to residuals;
  g. Evaluation of curve fitting efficacy to the residuals.

Result:

- The set of calibration functions for a given window width of MF, one for every determined section.

If the RMSE during the evaluation of the curve fitting is not satisfactory, the degree of the fitted regression line is increased, or the regression model is changed, and the curve fitting step is repeated.

The result of the above algorithm is the set of calibration functions, one for every determined section. It is possible to use these calibration functions only for specific ranges or to tabularise correction values for the whole calibrated range of the sensor. Correction values are coupled with the median filtration window width. MF is necessary with the window width used in the calibration process before the correction values can be applied.

(1) $$m_{c}=m_{r}-f_{c}(med(m_{r}))$$

Here,

- *m_c_* is the calibrated measurement;
- *m_r_* are the raw measurements;
- *f_c_* is the calibration function for a given range section;
- *med* is the median filtration function with a given window width.

## 4. The Research Results

The algorithm presented in Section 3 was used to determine the required correction data for the selected 2D LiDAR sensor. Initially, MF experiments were carried out with a window width of 3. Subsequently, tests were conducted to determine the optimal width of the filtering window, with the aim of achieving the highest possible precision in distance measurement. The resulting correction data were then processed using the MSC method with MF to achieve the highest possible accuracy in the distance measurement.

### 4.1. Experiments Using the Median Filter

**Figure 5:**
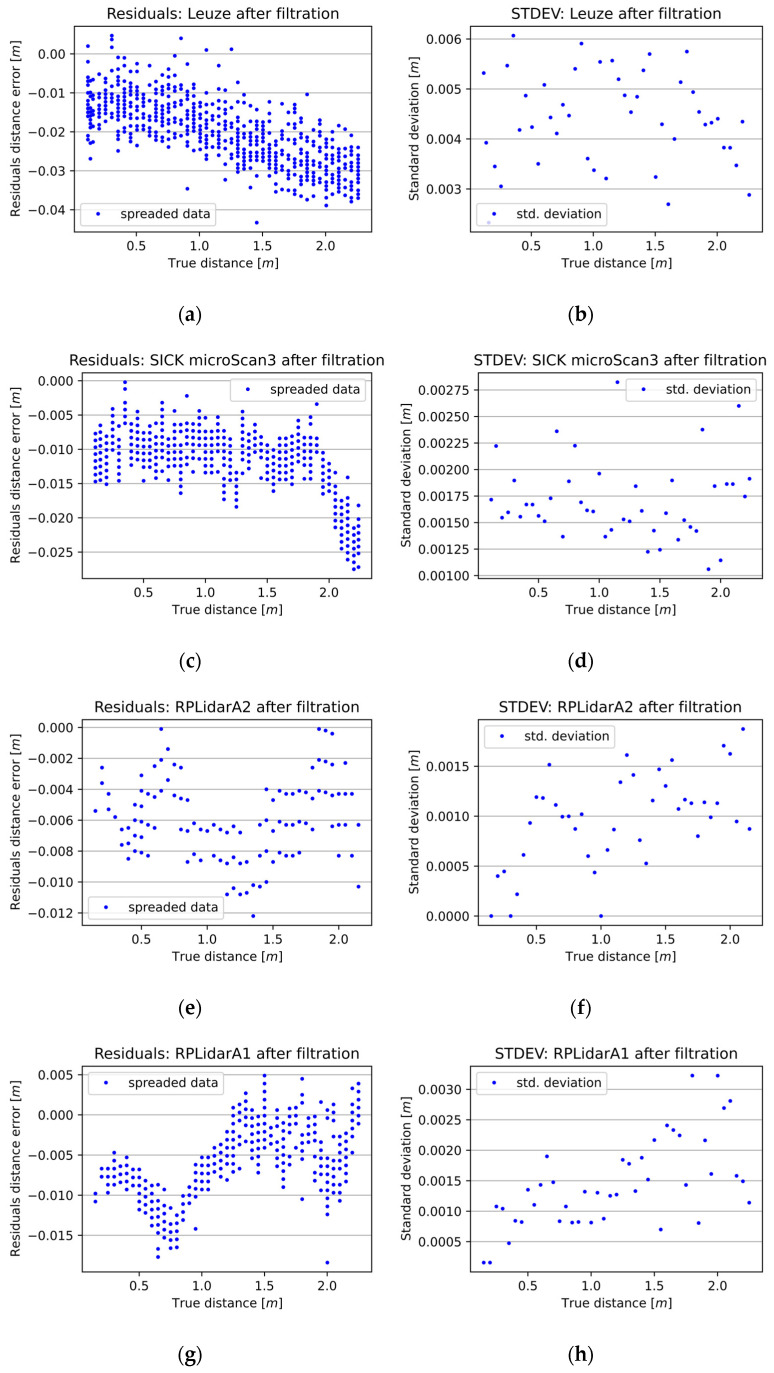
The outcome of 2D LiDAR measurements following the application of MF with a window width of 3. The left column (**a**,**c**,**e**,**g**) presents the differences between the 2D LiDAR sensors’ measurements and the reference distance measurements. The right column (**b**,**d**,**f**,**h**) presents the standard deviation of a given sensor at a given distance.

At the outset, median filtration experiments were conducted for the selected 2D LiDAR sensors with a window width of 3. In each case, it can be seen that the STDEV values decreased, and the measurement errors in each series for a given true distance shrank thanks to the MF (Figure 5). Finally, MF with a window width of 3 improved the precision measurements of the 2D LiDAR sensors from 21% for the RPLidarA2 to over 30% for the RSL425 XL, microScan3, and RPLidarA1.

**Figure 6:**
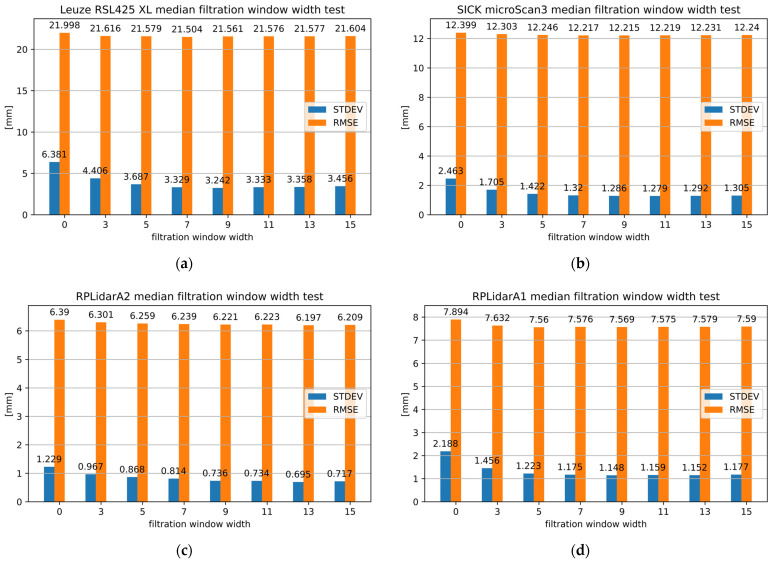
The results of 2D LiDAR measurements following the application of MF with different window widths for (**a**) RSL425 XL, (**b**) microScan3, (**c**) RPLidar A2 and (**d**) RPLidar A1.

Subsequent experiments were conducted to evaluate the influence of varying window widths on the precision and accuracy of the 2D LiDAR distance measurements. The research facilitated the identification of the optimal window width for each 2D LiDAR device under investigation. Figure 6 illustrates the influence of MF with a window width ranging from 0 to 15 on the measurements of the selected 2D LiDAR devices. The exact results of the MF study determining the optimal window width for the selected 2D LiDARs are presented in Table 5 in the “MF Imp” section.

In the context of 2D LiDAR safety, the optimal window width for the RSL425 XL was 9, which enabled a 49.19% improvement in precision. Consequently, the STDEV decreased from 0.00638 to 0.00324 m. However, the greatest improvement in accuracy, at 2.25%, was observed for a window width of 7.

The optimal window width for the microScan3 was 11, with a 48.07% improvement in precision. Consequently, the STDEV was reduced from 0.00246 to 0.00128 m. However, the greatest improvement in accuracy, amounting to 1.48%, was observed for the window width of 9.

For the RPLidarA1, the optimum window width was 9, with a precision improvement of 47.52%. As a result, the STDEV was reduced from 0.00219 to 0.00115 m. In this case, the best result in terms of increased accuracy of up to 4.23% was achieved with a window width of 5.

In the case of the RPLidar A2, the optimal window width was 13, with a 43.48% improvement in precision. Consequently, the STDEV was reduced from 0.00123 to 0.0007 m. In this instance, the most favorable outcome in terms of enhanced accuracy, up to 3.02%, was achieved with the same window width.

As can be observed, the utilization of MF, even at the smallest window width, has a significant impact on the enhancement of precision in 2D LiDAR measurements. However, it has a relatively limited influence on the accuracy of the measurements. Moreover, the selection of the optimal window width for precision does not necessarily guarantee the highest accuracy.

A reduction in precision can affect the accuracy of the results. Consequently, further experiments were conducted to investigate the influence of window width in the range from 0 to the optimal value.

### 4.2. Experiments of Multi-Section Calibration with Median Filtration

The test distance measurements of each 2D LiDAR sensor presented in Section 2, verified by the measurements of a certified Leica X4, were used for their MSC after the MF phase. These data can be calibrated by a series of fittings and a regression line to the distance measurements obtained. As described above, the calibration methods were applied to the four tested 2D LiDAR sensors.

**Figure 7:**
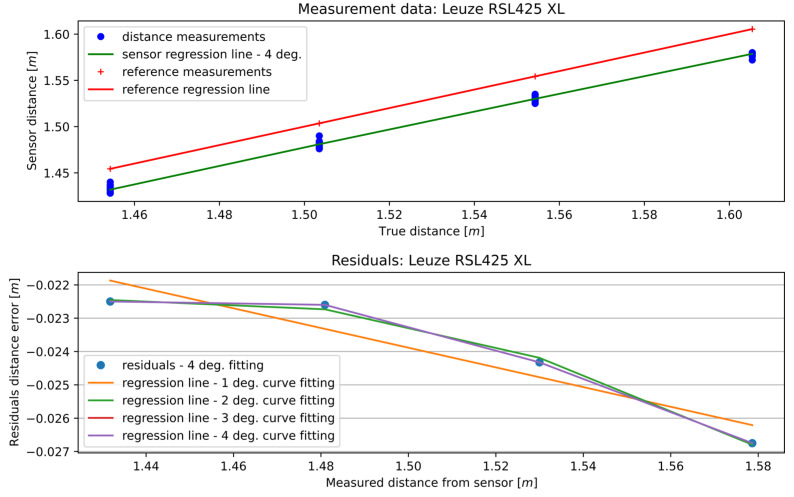
Curve fitting to the 13th section of the RSL425 XL sensor data—4th-degree polynomial regression line.

First, the MSCwMF method was applied to the RSL425 XL sensor. Figure 7 shows the results for different polynomial regression line degrees. At the top of the figure are the distance measurements gathered from the RSL425 XL sensor, together with the reference measurements and the regression line. There is also the result of curve fitting—the RSL425 XL’s polynomial regression line of a specific degree. At the bottom are the differences between the RSL425 XL and the reference regression lines. These points represent the correlation between the residual value and the distance measurement given by the RSL425 XL sensor. There are also polynomial regression lines of specific degrees fitted to the residuals. As can be observed, the accuracy of fitting increases with the degree of the fitted regression line. The higher the degree of regression line fitted to the measurements, the more characteristics of the sensor that can be fetched.

**Figure 8:**
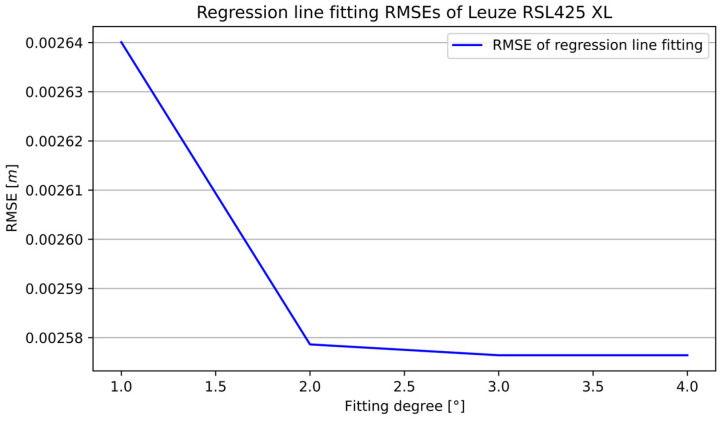
Evaluation of curve fitting to RSL425 XL sensor measurement data—RMSE.

Figure 8 presents the RMSE of the curve fitting to the distance measurements with different degrees of the fitted regression line. The smallest RMSE was given by the fourth-degree polynomial and equaled 0.002576 m.

**Figure 9:**
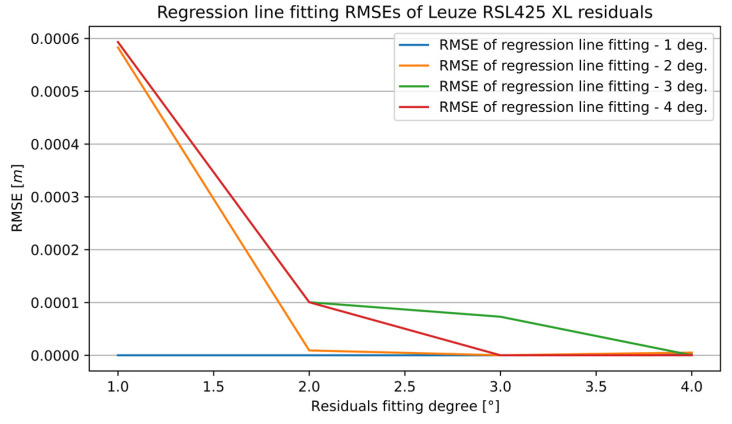
Evaluation of curve fitting to RSL425 XL sensor residuals—RMSE.

Figure 9 presents the RMSE of the curve fitting to the residuals with different degrees of the fitted polynomial regression line. Each line represents a specific degree of the curve fitting regression line to the distance measurements.

**Figure 10:**
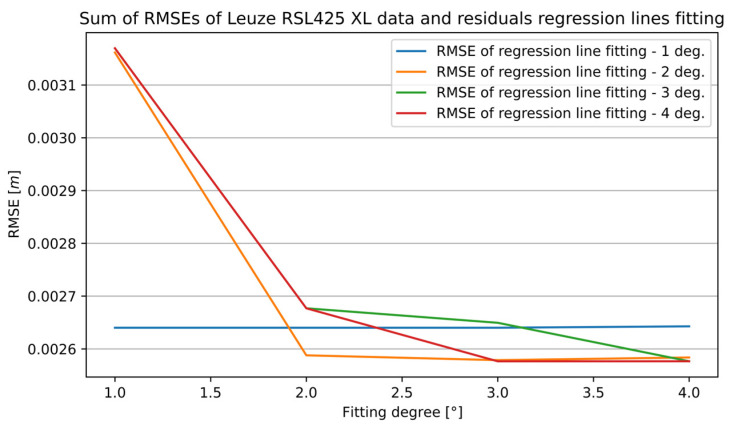
The sum of measurement data and residual curve fitting RMSEs of the RSL425 XL sensor for the 13th section.

The final accuracy of calibration depends on the two values of the RMSE calculated after curve fittings. That is why Figure 10 presents the sum of the RMSEs—the RMSE of the curve fitting to the distance measurements and the RMSE of the curve fitting to the residuals. The best accuracy measured by the RMSE for the RSL425 XL sensor is given by the pair of fitted regression lines of the fourth-degree polynomial for fitting to distance measurements and the third for fitting to the residuals.

Table 3 presents the pair of the best-fitted regression lines (to the distance measurements and residuals) with the parameters of their functions. Parameters of the function fitted to the residuals can be used to determine the sensor’s measurement errors correlated with the given distances and to calibrate the RSL425 XL sensor in a given range.

Such a calibration is performed for all the detected sections based on local minima and maxima residual points. Residuals were thus divided to extract measurement ranges with specific trends in data.

**Figure 11:**
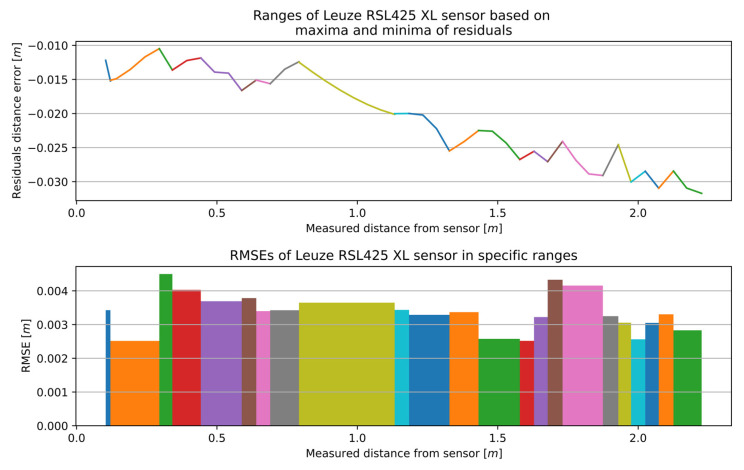
The outcome of the MSCwMF method for the RSL425 XL with a window width equal to 9 for MF. The same colour corresponds to the residual section generated using the regression line function fitted to individual segment and the RMSE value that best fit a specific section.

Figure 11 presents the results of the multi-section curve fitting calibration method. At the top of the figure are residual sections generated using regression line functions fitted to individual segments. At the bottom are the RMSE values that best fit a specific section.

**Figure 12:**
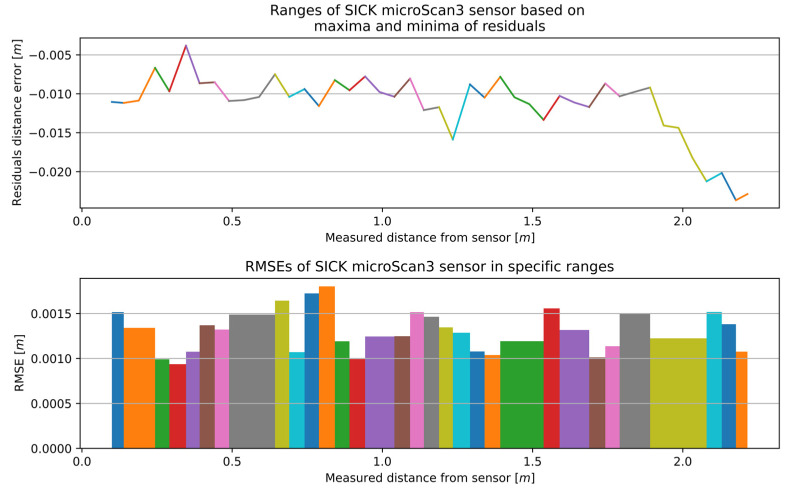
The outcome of the MSCwMF method for the microScan3 with a window width of 11 for MF. The same colour corresponds to the residual section generated using the regression line function fitted to individual segment and the RMSE value that best fit a specific section.

**Figure 13:**
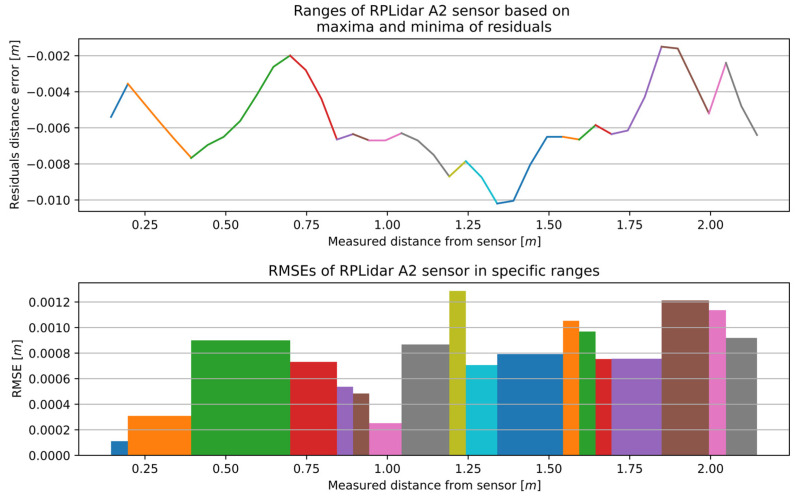
The outcome of the MSCwMF method for the RPLidar A2 with a window width of 13 for MF. The same colour corresponds to the residual section generated using the regression line function fitted to individual segment and the RMSE value that best fit a specific section.

**Figure 14:**
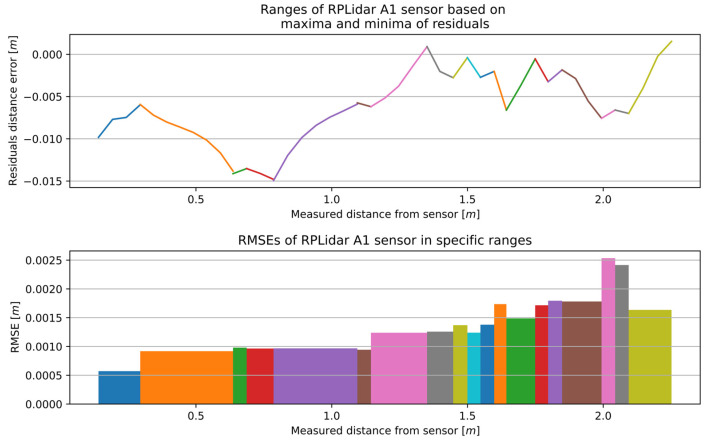
The outcome of the MSCwMF method for the RPLidar A1 with a window width of 9 for MF. The same colour corresponds to the residual section generated using the regression line function fitted to individual segment and the RMSE value that best fit a specific section.

The aforementioned calibration method for the RSL425 XL was implemented on the microScan3, RPLidar A2, and RPLidar A1. The characteristics for the remaining 2D LiDAR sensors with optimal window width are presented in Figure 12, Figure 13 and Figure 14, while the number of sections and the range of RMSE values are presented in Table 4. The RMSE value of the best fitting was calculated for each section. The final RMSE value for the MSC was calculated based on the average RMSE values from all sections.

The complete measurement range for the RSL425 XL was partitioned into 23 sections, and the accuracy ranged between 0.00251 and 0.00450 m.

The microScan3 had the most defined individual segments, and the accuracy ranged from 0.00094 to 0.00180 m. The 32 sections contributed to a stable improvement in accuracy over the entire distance measurement tested. Furthermore, the smallest difference between the minimum and maximum RMSE values was observed in this case.

The complete measurement range for the RPLidar A2 was divided into 18 sections, with an accuracy range of 0.00011 to 0.00129 m. Similarly, the complete measurement range for the RPLidar A1 was divided into 19 sections, with an accuracy range of 0.00057 to 0.00253 m.

Dividing the whole range into the smallest and monotonic sections introduces a high sensitivity to the deviation of measurements. Each section is handled by a separate function, and the steep functions that covered a narrow measurement range with a high amplitude of residuals were the weak points of the multi-section curve fitting.

### 4.3. Comparison of Usage of the MSCwMF Method for Selected 2D LiDAR

The aforementioned MSCwMF method was employed in the analysis of selected 2D LiDAR data for window widths ranging from 0 to optimal. The outcomes of the calibration methodology for the selected 2D LiDAR sensors are presented in Table 5. The initial RMSE and STDEV values of the raw data, prior to filtering and calibration, are presented for each 2D LiDAR sensor in a row for a window width of 0.

The incremental improvements of the MSCwMF method, achieved by increasing the MF window width and further processing with the MSC method, were compared to the RMSE and STDEV values for a window width of 0 (no filtering). The results of this comparison for the selected 2D LiDARs are presented in Table 5, in sections “MSCwMF” and “IncrImp”. The optimal window width for the RSL425 XL was 9, which enabled precision improvements of 48.99%. This resulted in a reduction of the STDEV from 0.00645 to 0.00329 m. In this instance, the most favorable outcome in terms of enhanced accuracy of 47.55% was achieved. Consequently, the RMSE was reduced from 0.00652 to 0.00342 m. For the microScan3, the optimal performance was observed with a window width of 11 units, leading to precision improvements of 48.19%. The STDEV decreased to 0.00129 m from 0.00249 m. This allowed for accuracy enhancements of up to 47.24%, resulting in a reduction in RMSE values from 0.00254 to 0.00134 m. In the case of the RPLidar A2, the optimal window size was 13, which resulted in a precision improvement of approximately 43.90%. This reduced the STDEV to 0.00069 m from 0.00123 m. This solution ensured accuracy enhancements of up to 39.10% and reduced the RMSE value from 0.00133 to 0.00081 m. Lastly, for the RPLidar A1, with an optimal window width of 9, precision improvements were recorded at 47.49%. The STDEV was reduced from 0.00218 to 0.00114 m. The accuracy outcomes improved to 46.69%, and RMSE values were lowered from 0.00244 m to the more accurate figure of 0.00130 m.

As can be observed, in all cases involving the MSCwMF method, the STDEV value decreased as the window width increased to its optimal level, resulting in a corresponding reduction in the RMSE.

### 4.4. Impact of the MSCwMF Method on the Improvement of Measurement Accuracy

In Table 5, the “Imp” section presents a comparative analysis of the overall improvement in the 2D LiDAR measurements of precision and accuracy for different window widths using the MSCwMF method.

In the case of the RSL425 XL, the absence of MF (window width 0 for the sensor before calibration) resulted in a notable enhancement in measurement accuracy, with the MSCwMF method contributing to an overall improvement of over 70%. Utilization of the optimal window width of nine improved the precision by over 48% and accuracy exceeding 84%. Therefore, the application of MF for an optimal window width in conjunction with MSC increased accuracy by over 14%. Consequently, the RMSE was reduced from 0.022 to 0.00342 m, and STDEV was reduced from 0.00638 to 0.00329 m.

For the microScan3 sensor, the MSCwMF method resulted in an improvement of more than 79%. When a window width of 11 was employed, precision improvements exceeded 47%, and accuracy advancements reached above 89%. In this case, the accuracy increase was nearly 10%. As such, the RMSE was reduced from 0.0124 to 0.00134 m, while the STDEV decreased from 0.00246 to 0.00129 m.

The RPLidar A2 sensor also benefited significantly from the MSCwMF method, which contributed to an overall improvement surpassing 79%. When the optimal window width was set at 13, precision improvements exceeded 43%, and accuracy enhancements exceeded 87%. In this case, the accuracy increase was above 8%. This led to a reduction in the RMSE from 0.00639 to 0.00081 m and a decrease in STDEV from 0.00123 to 0.00069 m.

For the RPLidar A1 sensor, the use of the MSCwMF method resulted in an overall improvement exceeding 69%. With an optimal window width of nine, precision improvements exceeded 47%, and accuracy enhancements surpassed 83%, with accuracies increasing by over 14%. The RMSE was reduced from 0.00789 to 0.0013 m, and the STDEV decreased from 0.00219 to 0.00114 m.

The results obtained were consistent for all window widths tested, demonstrating the effectiveness of the methodology developed for all selected 2D LiDAR devices. The MSCwMF method using an optimal window width ensured a high level of accuracy, surpassing 89%. The MF method increased the precision to over 48%. The comparison of MSCwMF with optimal window width to no filtering showed an improvement of more than 14%, demonstrating the importance of using MF in the overall method.

A comparison of the results obtained after filtration and after filtration combined with MSC for the same window width for the selected 2D LiDAR devices revealed that the STDEV value exhibited only a slight difference in most cases, whereas the RMSE value demonstrated a notable reduction. It can thus be concluded that the application of MSC has a marginal impact on reducing precision while markedly enhancing accuracy.

## 5. Conclusions

In the presented study, the authors undertook a comparative analysis of the measurement characteristics of four 2D LiDAR systems to identify potential avenues for enhancing the precision and accuracy of their distance measurements.

During the study, it was shown that the proposed calibration method significantly increases the accuracy of 2D LiDAR measurements, while the filtering method increases the precision of such measurements. The partitioning of the complete measurement range into sections resulted in a notable enhancement in sensitivity to deviations in measurements. The application of MF, aimed at identifying an optimal window width in conjunction with MSC, was demonstrated to significantly enhance accuracy. Furthermore, the proposed calibration method enhanced the accuracy of the sensors to a degree nearly equivalent to their STDEV.

The optimum window width for MF is most useful for verifying the position of the AGV when it is stationary, e.g., at intermediate points or after docking. In such cases, the MSCwMF method provides the most accurate distance measurements and should be used with MSC, with the selected optimum value of the window width set for the filter phase.

In contrast, MF should not be used when the AGV is in motion. In such cases, the MSCwMF method still provides good-quality, accurate distance measurements and should be used with MSC, with the window width value set to zero for the MF (eliminating the filtration phase). It should be noted that the choice of filter window width depends on the ability to select the angular velocity of the specific 2D LiDAR device for a given AGV speed. In fact, it depends on the possible high frequency of measurements provided by 2D LiDAR systems. As a result, even when measuring for a window of width three, the AGV will move a certain distance. To compensate for this displacement from the 2D LiDAR measurements, it is necessary to use measurements from other sensors (e.g., an encoder). This introduces an additional source of measurement error. Achieving such high measurement accuracy using additional sensors is, therefore, quite difficult. An alternative solution is using a different filtering method to remove outliers from the 2D LiDAR measurements. In effect, both solutions require further research to achieve a measurement quality close to that of MF when the AGV is in a standing position.

The efficacy of the proposed MSCwMF method was demonstrated through empirical research. It was established that implementing MF results in a reduction in the number of calibrated measurements. Consequently, a proportion of the information extracted from 2D LiDAR is forfeited. However, the research demonstrated that the application of MF prior to MSC can enhance measurement accuracy by up to 14% in comparison with the absence of MF.

The proposed MSCwMF method has a significant impact on the precision and accuracy of distance measurements for both classes of safety and range 2D LiDAR systems. In fact, the proposed MSCwMF method can be used to generate several sets of correction values with different window widths for MF. Depending on the selected driving mode or action, the appropriate set of correction values with the corresponding width of the filter window can be selected. The findings suggest that the developed method may have broader applicability beyond the initial scope of this study, potentially offering utility in other 2D LiDAR solutions.

## Figures and Tables

**Table 1:** The comparison of the selected 2D LiDAR sensors.

LiDAR Model	Range	Resolution	Measurement Error	FOV	Angular Resolution	Frequency
RSL425 XL	0.05–50 m	0.001 m	0.01–0.02 m	270 deg	0.1 deg	25 Hz
microScan3	0.1–64 m		0.025 m	275 deg		
RPLidar A1	0.15–12 m	0.005 m < 1.5 m and 1% > 1.5 m	<1%	360 deg	1 deg	5.5 Hz
RPLidar A2	0.15–16 m				0.9 deg	8 Hz
Leica DISTO X4	0.05–150 m	0.0001 m	0.001 m < 10 m	0.034 deg	0.034 deg	7 Hz

**Table 2:** Calculated statistical information about 2D LiDAR sensors.

Sensors	Standard Deviation [m]				Residuals [m]			
	Min.	Avg.	Max.	Max–Min	Min.	Avg.	Max.	RMSE
RSL425 XL	0.0047	0.0064	0.0081	0.0034	−0.0520	−0.0200	0.0087	0.022
microScan3	0.0017	0.0025	0.0038	0.0021	−0.0285	−0.0114	0.0009	0.0124
RPLidar A1	0.0002	0.0022	0.0053	0.0051	−0.0204	−0.0063	0.0083	0.0079
RPLidar A2	0 *	0.0012	0.0020	0.0020	−0.0124	−0.0059	0.0018	0.0064

* The parameter of the min. STDEV for the RPLiDAR A2 (calculated from the test measurements) equals a value that is lower than the measurement resolution of the tested device.

**Table 3:** Set of the best 13th section curve fitting regression lines with their parameters—RSL425 XL sensor.

	Range		Best Fitting Degree	Parameters				
13th Section Curve Fitting Regression Lines	From	To		a	b	c	d	e
Reference measurement data	1.4543	1.6054	4	0.3834	−1.1095	−0.5180	4.8484	−2.8263
Residuals	1.4318	1.5787	3	1.2748	−6.0009	9.3645	−4.8704	-

**Table 4:** The number of sections and the range of RMSE values for the optimal window width.

Sensor	Number of Sections	RMSE [m]		
		Min	Max	Difference
RSL425	23	0.00251	0.00450	0.00198
microScan3	32	0.00094	0.00180	0.00087
RPLiDAR A2	18	0.00011	0.00129	0.00118
RPLiDAR A1	19	0.00057	0.00253	0.00196

**Table 5:** The outcomes of the calibration methodology for the selected 2D LiDAR sensors.

**RSL425 XL**	MF Imp *				MSCwMF **		IncrImp ***		Imp ****	
**Window width**	**STDEV [m]**	**RMSE [m]**	**STDEV imp**	**RMSE imp**	**STDEV [m]**	**RMSE [m]**	**STDEV imp**	**RMSE imp**	**STDEV imp**	**RMSE imp**
0	**0.00638**	**0.02200**	0.00%	0.00%	**0.00645**	**0.00652**	0.00%	0.00%	−1.08%	70.36%
3	0.00441	0.02162	30.95%	1.74%	0.00448	0.00459	30.54%	29.60%	29.79%	79.13%
5	0.00369	0.02158	42.22%	1.90%	0.00373	0.00386	42.17%	40.80%	41.55%	82.45%
7	0.00333	***0.02150***	47.83%	***2.25%***	0.00337	0.00351	47.75%	46.17%	47.19%	84.04%
***9***	***0.00324***	0.02156	***49.19%***	1.99%	***0.00329***	***0.00342***	***48.99%***	***47.55%***	***48.44%***	***84.45%***
**microScan3**	MF Imp *				MSCwMF **		IncrImp ***		Imp ****	
**Window width**	**STDEV [m]**	**RMSE [m]**	**STDEV imp**	**RMSE imp**	**STDEV [m]**	**RMSE [m]**	**STDEV imp**	**RMSE imp**	**STDEV imp**	**RMSE imp**
0	**0.00246**	**0.01240**	0.00%	0.00%	**0.00249**	**0.00254**	0.00%	0.00%	−1.10%	79.51%
3	0.00171	0.01230	30.78%	0.77%	0.00173	0.00177	30.52%	30.31%	29.76%	85.72%
5	0.00142	0.01225	42.27%	1.23%	0.00144	0.00149	42.17%	41.34%	41.53%	87.98%
7	0.00132	0.01222	46.41%	1.47%	0.00134	0.00138	46.18%	45.67%	45.59%	88.87%
9	0.00129	***0.01222***	47.79%	***1.48%***	0.00130	0.00135	47.79%	46.85%	47.22%	89.11%
***11***	***0.00128***	0.01222	***48.07%***	1.45%	***0.00129***	***0.00134***	***48.19%***	***47.24%***	***47.62%***	***89.19%***
**RPLidar A2**	MF Imp *				MSCwMF **		IncrImp ***		Imp ****	
**Window width**	**STDEV [m]**	**RMSE [m]**	**STDEV imp**	**RMSE imp**	**STDEV [m]**	**RMSE [m]**	**STDEV imp**	**RMSE imp**	**STDEV imp**	**RMSE imp**
0	**0.00123**	**0.00639**	0.00%	0.00%	**0.00123**	**0.00133**	0.00%	0.00%	−0.08%	79.19%
3	0.00097	0.00630	21.32%	1.39%	0.00097	0.00107	21.14%	19.55%	21.07%	83.26%
5	0.00087	0.00626	29.37%	2.05%	0.00087	0.00098	29.27%	26.32%	29.21%	84.66%
7	0.00081	0.00624	33.77%	2.36%	0.00081	0.00092	34.15%	30.83%	34.09%	85.60%
9	0.00074	0.00622	40.11%	2.64%	0.00073	0.00086	40.65%	35.34%	40.60%	86.54%
11	0.00073	0.00622	40.28%	2.61%	0.00073	0.00085	40.65%	36.09%	40.60%	86.70%
***13***	***0.00070***	***0.00620***	***43.45%***	***3.02%***	***0.00069***	***0.00081***	***43.90%***	**39.10%**	***43.86%***	***87.32%***
**RPLidar A1**	MF Imp *				MSCwMF **		IncrImp ***		Imp ****	
**Window width**	**STDEV [m]**	**RMSE [m]**	**STDEV imp**	**RMSE imp**	**STDEV [m]**	**RMSE [m]**	**STDEV imp**	**RMSE imp**	**STDEV imp**	**RMSE imp**
0	**0.00219**	**0.00789**	0.00%	0.00%	**0.00218**	**0.00244**	0.00%	0.00%	0.49%	69.13%
3	0.00146	0.00763	33.46%	3.32%	0.00145	0.00163	33.43%	33.31%	33.75%	79.41%
5	0.00122	***0.00756***	44.09%	***4.23%***	0.00122	0.00138	44.07%	43.55%	44.34%	82.57%
7	0.00117	0.00758	46.30%	4.03%	0.00117	0.00133	46.24%	45.28%	46.50%	83.11%
***9***	***0.00115***	0.00757	***47.52%***	4.12%	***0.00114***	***0.00130***	***47.49%***	***46.69%***	***47.74%***	***83.54%***

* The results of the MF improvement process. Bold indicates the values obtained from the raw measurements and italics indicates the best values obtained after filtering for selected 2D LiDAR. In the following cases, italics indicate the best values obtained after filtering with a given window width and calibration. ** The results of the MSCwMF improvement process. Bold indicates values obtained from calibrated measurements without filtering. *** The results of the MSCwMF incremental improvement process after increasing the MF window width. **** The overall improvement of the MSCwMF method.

## Data Availability

The original contributions presented in this study are included in the article. Further inquiries can be directed to the corresponding author.
